# Evaluation of a detomidine–ketamine–azaperone combination for the chemical immobilization of fallow deer (*Dama dama*)

**DOI:** 10.3389/fvets.2025.1718243

**Published:** 2025-11-13

**Authors:** Giulia Maria De Benedictis, Alice Baggio, Stefano Pesaro, William Magnone, Giovanna Miani, Alice Andolfatto, Lucia Bono, Francesca Zanusso

**Affiliations:** 1 Department of Animal Medicine, Production and Health, University of Padova, Padova, Italy; 2 Department of Agricultural, Food, Environmental and Animal Sciences, University of Udine, Udine, Italy; 3 Independent Practitioner, Milan, Italy; 4 Department of Ecology and Emergence of Zoonotic Diseases, Helmholtz Institute for One Health, Greifswald, Germany; 5 Independent Practitioner, Turin, Italy; 6 Parco Faunistico Cappeller, Cartigliano, Italy

**Keywords:** fallow deer, chemical immobilization, detomidine, azaperone, ketamine, quality assessment

## Abstract

The fallow deer (*Dama dama*) is a widely distributed cervid in Europe, often managed in enclosed settings for conservation, education, or hunting purposes. Chemical immobilization is frequently required during routine handling; however, fallow deer remain understudied in the scientific literature with regard to standardized anesthetic protocols. This study aimed to evaluate the efficacy and safety of an anesthetic protocol combining detomidine (0.2 mg/kg), ketamine (2 mg/kg), and azaperone (0.3 mg/kg) in 22 captive fallow deer. Animals were immobilized for clinical procedures, including health checks, blood sampling, individual identification, and translocation. The quality of anesthesia and physiological parameters (heart rate, respiratory rate, oxygen saturation, end-tidal CO_2_, arterial blood pressure, temperature) were monitored throughout immobilization. Anesthetic depth was evaluated through the assessment of reflexes, muscle tone, and eye position. The quality of induction, immobilization at approach, and recovery were systematically evaluated using dedicated scoring systems. Induction was smooth in all animals, with lateral recumbency time, and time to first safe approach achieved in 10.7 ± 6.4 min, and 22.0 ± 5.1 min, respectively. Mean handling time was 49.8 ± 9.6 min. Physiological parameters remained within acceptable limits, with only transient hypoxemia. Recovery was uneventful in most individuals, with the majority exhibiting good to excellent recovery quality and standing within 20 min of atipamezole administration. Only one individual experienced a poor-quality and prolonged recovery. No complications were observed post-procedure. The detomidine-ketamine-azaperone combination proved to be an effective and safe protocol for immobilizing fallow deer, providing stable anesthesia and good recovery quality. Dedicated scoring systems enabled a standardized and repeatable evaluation of the quality of the used protocol and its suitability. This study is the first to describe the use of azaperone in combination with detomidine and ketamine in fallow deer, contributing new insights into non-opioid immobilization strategies for cervids.

## 1 Introduction

The fallow deer (*Dama dama*) is a widely distributed, naturalized species in Europe, commonly managed in fenced environments such as parks, zoos, and game reserves for conservation, educational, and hunting purposes (1, 2). Population management interventions, including translocations, clinical assessments or microchipping for individual identification, often require chemical immobilization to ensure the safety of both animals and personnel involved (3–5). However, fallow deer are highly sensitive to stress, making them particularly prone to capture-related complications such as respiratory depression, bloat, regurgitation, and potentially fatal capture myopathy (6–8).

Various drug protocols have been tested in cervids, including ultra-potent opioids like etorphine or thiafentanil, often combined with sedatives, to improve immobilization quality. Nonetheless, these combinations are associated with severe adverse effects, including dysrhythmias, hypoxemia, apnea, muscle rigidity, and prolonged recoveries (6, 9). Safer alternatives, such as α_2_-agonists (e.g., xylazine, detomidine, medetomidine) combined with dissociative agents like ketamine or tiletamine/zolazepam, have been explored (7, 10), but reports often lack standardized physiological monitoring or consistent dosing strategies.

Importantly, α_2_-agonists exert dose-dependent depressant effects on respiration (11), suggesting that dose optimization may mitigate risks while maintaining effective sedation. Although fallow deer are increasingly targeted for chemical immobilization, they remain underrepresented in the scientific literature. This highlights the need for further evaluation of non-opioid anesthesia protocols to establish safe and reliable immobilization strategies for this species.

The aim of this study was to evaluate the efficacy and safety of a non-opioid-based anesthetic protocol combining detomidine, ketamine, and azaperone in captive fallow deer. Specifically, we sought to:

- evaluate the clinical parameters during immobilization, including heart rate, respiratory rate, hemoglobin oxygen saturation, end-tidal CO_2_, rectal temperature, and arterial blood pressure;
- assess the quality of induction, immobilization, and recovery to determine the adequacy of sedation for conducting non-invasive procedures without supplemental dosing;
- identify any adverse effects or complications arising during immobilization, such as respiratory depression, hypoxemia, muscle rigidity, or prolonged recovery, and evaluate their clinical relevance.

It was hypothesized that the combination of detomidine, ketamine and azaperone would be an effective and safe protocol for the pharmacological immobilization of fallow deer.

## 2 Materials and methods

### 2.1 Animals

This study was approved by the Animal Welfare Body (OPBA) of the University of Padova (Protocol no. 80/2023), and all procedures complied with national ethical and professional standards. Chemical immobilizations of fallow deer were performed as part of routine management procedures, including health evaluations, blood sampling, individual identification via microchip and ear tag application, and translocation to new facilities. The study included captive fallow deer (*Dama dama*) of both sexes and various age groups, immobilized at three locations in Northern Italy. Immobilization sessions were conducted between February and April 2024 and in February 2025. The first location was the Antonio Servadei Farm of the University of Udine (46.12571° N, 13.17753° E, 169.00 m a.s.l.), where deer roamed within a ~20-hectare naturalistic enclosure. The second location was the Agricultural Institute Pastori, Brescia (45.52694° N, 10.261464° E, 132.00 m a.s.l.), which housed deer in a ~400 m^2^ outdoor enclosure with access to a covered shelter, trees, and a central pond. The third location was La Fodoma Farm, Chions (45.88861° N, 12.76589° E, 16,00 m a.s.l.), where a group of fallow deer lived on approximately 500 m^2^ of land. During the sessions, ambient temperatures ranged between 12 °C and 20 °C.

All deer lived in social groups and had *ad libitum* access to pasture grass, hay and water. Animals were selected for immobilization based on visible indicators of good physical condition, including normal posture, coat quality, and body condition. Individuals showing signs of poor health, abnormal behavior, or very young age were excluded from the study.

Animals were classified into age groups based on estimated age, body size, secondary sexual characteristics, dental wear, presence and size of antlers as follows: yearlings (approximately 1 year old), young adults (2–3 years), and adults (≥3 years).

### 2.2 Anesthesia protocol

Chemical immobilization was achieved using an intramuscular combination of detomidine (Sedaquick, 10 mg/mL, Fatro, Italy) 0.2 mg/kg, ketamine (Ketavet 100, 100 mg/mL, Intervet Productions Srl, Italy) 2 mg/kg, and azaperone (Suiwell, 40 mg/mL, ATI Azienda Terapeutica Veterinaria, Italy) 0.3 mg/kg. Animals were chemically immobilized without prior separation, remaining within their social group to minimize handling-related stress. The drugs were delivered remotely using a CO_2_-powered dart rifle (DAN-INJECT CO_2_ injection rifle, model J.M.SP. DAN-INJECT ApS, Børkop, Denmark) and 3-mL dart syringe with 2.0 x 30 mm collared needle with side ports (Dan-Inject, Børkop, Denmark). The distance to the animal, the CO_2_ pressure used for dart propulsion, and the anatomical site of dart impact were recorded. Drug dosages were calculated based on estimated body weight assessed by two experienced veterinarians (SP, WM).

A supplemental intramuscular dose was administered if adequate sedation was not achieved within 15 min due to incomplete drug effect or suspected dart failure.

The induction phase was systematically monitored by evaluating behavioral and postural indicators of sedation, with particular attention to the time required to attain stable lateral recumbency. Following drug administration, fallow deer were left undisturbed and continuously monitored throughout the induction period. Key timepoints were recorded from the moment of drug administration, including:

- ataxia time (onset of incoordination);
- sternal recumbency time (animal assuming a sternal position);
- lateral recumbency time (animal lying on its side);
- approach time.

Once the animal was in lateral recumbency and unresponsive to external stimuli, two experienced veterinarians proceeded with the approach. At this stage, the animal was blindfolded to minimize external stimuli, and transferred outside the enclosure to a more accessible, shaded area protected from radiant heat, where the scheduled procedures were carried out.

During the maintenance phase, the animal was continuously monitored. All animals underwent a complete physical and dental examination to assess their clinical condition. The body condition score (BCS) was also assessed using a 5-point scale (12). A blood sample was collected from each animal during immobilization for complete hematological and biochemical analysis. An abdominal ultrasound examination was also performed, primarily to evaluate the pregnancy status of the animals.

A jugular intravenous (IV) catheter 14G (Introcan Safety^®^ 14G, 50 mm, B. Braun, Germany) was aseptically inserted. During immobilization, deer were maintained in sternal or in lateral recumbency with the neck elevated above the level of the rumen and the muzzle directed toward the ground to promote airway patency and minimize the risk of regurgitation or aspiration. Artificial tear drops were administered every 15 min to maintain ocular lubrication throughout the procedure. Propofol (Proposure 1%, Boehringer Ingelheim Animal Health Italia S.p.A., Italy) was available as rescue anesthesia at a dose of 1 mg/kg IV in cases where the depth of anesthesia was deemed insufficient, to ensure safety throughout the procedure.

At the end of the procedure, each animal was weighed using a digital dynamometer (Sauter FH 100, Sauter GmbH, Balingen, Germany) and subjects scheduled for translocation were placed in individual transport crates.

Eventually, recovery began with the intramuscular administration of atipamezole (Antisedan, 5 mg/mL, Vetoquinol Italia Srl, Italy) at a dose of 0.2 mg/kg, calculated based on the initial estimated body weight and administered at the end of the procedures. The following recovery times were recorded:

- time to first movements;
- time to sternal recumbency;
- time to standing position.

Handling time was defined as the time from the approach to the administration of atipamezole.

Actual body weight was recorded to retrospectively calculate the exact drug doses administered.

### 2.3 Clinical monitoring

Throughout the immobilization period, animals were continuously monitored, and their physiological parameters were recorded at 5-min intervals. Time zero (T0) corresponded to approach to the animal, followed by T5, T10, and subsequent time points.

Assessed clinical parameters included:

- heart rate (HR) via cardiac auscultation and cross-checked with a pulse oximeter (VE-H100B, Edan Instruments Inc., Germany) placed on the tongue;
- respiratory rate (RR) by visual observation of thoracic movements;
- hemoglobin oxygen saturation (SpO_2_) using the same pulse oximeter;
- end-tidal CO_2_ (ETCO_2_) via a portable capnograph (Emma™ Capnograph, Masimo, Switzerland) positioned at one nostril;
- arterial blood pressure (systolic SAP, diastolic DAP, mean MAP) using an oscillometric device (PetTrust, BioCare, Taiwan), with a cuff placed at the tail base, selected according to tail circumference;
- rectal temperature (Temp) using a digital thermometer.

The depth of anesthesia was assessed by evaluating palpebral and anal reflexes, muscle tone, and eye position, with corresponding scores assigned to these parameters (Table 1).

**Table 1:** Scoring system used to assess the depth of anesthesia during the chemical immobilization of fallow deer.

**Parameter**	**0**	**1**	**2**	**3**
Muscle tone	Absent	Decreased	Normal	
Palpebral reflex	Absent	Weak	Strong	Spontaneous
Anal reflex	Absent	Decreased	Normal	
Eye position	Ventro-medial	Partially ventro-medial	Central	

Additionally, fallow deer were monitored for signs of rumen distention and bloat visually. Measurement of blood lactate and glucose levels took place once in each animal during immobilization using handheld analyzers (Accu-Chek Guide Glucometer, Roche Diabetes Care GmbH, Germany; Lactate Scout 4, EKF Diagnostics, United Kingdom).

### 2.4 Anesthesia quality assessment

Dedicated scoring systems were developed specifically to evaluate the quality of induction, immobilization at approach, and recovery.

Induction quality was rated on a scale from 0 to 3 (Table 2), where 0 indicated poor and 3 excellent induction. The score was evaluated from the time of drug administration up to just before the approach to the animal.

**Table 2:** Scoring system used to assess the quality of induction during the chemical immobilization of fallow deer.

**Score**	**Description**
0—Poor	Rough and lengthy induction, severe ataxia, not recumbent within 15 min
1—Moderate	Rough induction, severe ataxia, recumbent after numerous attempts
2—Good	Relatively rapid and smooth induction, moderate ataxia, recumbent in 2 attempts
3—Excellent	Rapid and smooth induction, slight ataxia, no signs of excitement, smooth transition to lateral recumbency

Immobilization at approach was scored from 0 to 6 (Table 3), with 0 representing no visible effect and 6 indicating excessive immobilization.

**Table 3:** Immobilization score at approach after administration of a combination of detomidine-ketamine-azaperone in fallow deer.

**Score**	**Description**
0—no effect	Maintained ability to rise and walk in an ungainly manner, normal movement, normal ear and neck position, normal posture.
1—minimal	Drooping eyelids, lightly decreased frequency and rapidity of movement, lowered ear and head position, lip drooping, slightly relaxed postural tone (low head carriage, ataxia), animal moves away when approached.
2—mild	Low head carriage, ataxia, animal does not move away when approached and can be touched; standing with moderate ataxia, braced stance, and sometimes lowered head.
3—moderate	Sternal recumbency with head up, will attempt to rise when stimulated, difficult handling, spontaneous motor activity, presence of anal or palpebral reflexes, responsive to painful stimuli.
4—deep	Lateral recumbency, slight response to stimuli (eye or ear movement), but inability to lift head; unable to maintain an erect head, and noticeable eye or ear movement, will not attempt to rise when stimulated.
5—very deep	Prolonged periods of immobility, loss of postural tone, sternal or lateral recumbency with no sign of reversal (sternal recumbency with head down, does not lift head on stimulation, can be rolled into lateral recumbency and maintains this position); complete immobilization without response to any stimuli (sound, touch). Smooth, complete relaxation, extractable tongue, loss of pedal reflex, no involuntary tail movements, no reaction to blood sampling, safe handling.
6—excessively deep	Smooth, complete relaxation, extractable tongue, loss of palpebral reflex and jaw tone, no involuntary tail movements, no reaction to blood sampling, safe handling.

Recovery quality was graded on a scale from 0 (very poor) to 5 (excellent) (Table 4).

**Table 4:** Scoring system used to assess the quality of recovery after administration of atipamezole in fallow deer.

**Score**	**Description**
0—Very poor	Lateral recumbency 30 min after administration of antagonist, not responsive.
1—Poor	Agitated recovery, marked ataxia, high risk of injury.
2—Fair	Severe ataxia, numerous attempts to stand, frequent falls.
3—Moderate	Prolonged ataxia, some attempts to reach standing position.
4—Good	Moderate ataxia, more than two attempts to reach standing position.
5—Excellent	Minimal ataxia, one or two attempts to reach standing position.

At the end of each immobilization procedure, the attending veterinarians collectively discussed the scores to ensure consistency in evaluation.

### 2.5 Adverse effects and complications

Throughout the immobilization period, animals were continuously monitored for the occurrence of adverse effects or complications. Respiratory depression was defined as a respiratory rate below a predefined threshold (10 breaths/min), while hypoxemia was identified as a peripheral oxygen saturation (SpO_2_) below 90%. Potential re-sedation was monitored for 6 h post-immobilization. All behavioral and physiological responses potentially indicative of distress or drug-related side effects were documented.

Emergency equipment, including an oxygen source, endotracheal intubation devices, emergency drugs and fluids, was immediately available. Oxygen was ready to be administered in cases of hypoxemia and respiratory depression. For abdominal bloating, a rumen tube and trocar were available for decompression if abdominal massage failed to induce belching.

### 2.6 Statistical analysis

The Shapiro-Wilk test was used to assess the normality of continuous variables. Depending on data distribution, continuous variables were presented as mean ± standard deviation (SD) or median with interquartile range (IQR). Categorical variables were summarized as absolute counts and percentages.

Data analysis was performed using linear mixed models to investigate the effect of time on various clinical variables in the animals. The parameters analyzed for each individual included: HR, RR, SpO_2_, ETCO_2_, SAP, MAP, DAP, and Temp, all recorded at 5-min intervals starting from the approach (T0) to 40 min post-approach (T40).

For each clinical variable, a linear mixed-effects model was applied, with time as the fixed effect and individual animal as a random effect. This approach allowed for the assessment of temporal trends in each parameter while accounting for inter-individual variability.

All statistical analyses were conducted using R Studio (RStudio, PBC, Boston, MA, US) as interface for R (The R Foundation for Statistical Computing, Austria). A *p*-value < 0.05 was considered statistically significant.

## 3 Results

### 3.1 Animals

A total of 22 European fallow deer (*Dama dama*), comprising 12 males and 10 females, were included in the study. Two males were immobilized at the Antonio Servadei Farm, 15 fallow deer were housed at the Agricultural Institute, and 5 animals at La Fodoma Farm.

The study included 3 yearling males, 3 young adult males, 6 adult males, 3 yearling females, and 7 adult females. The BCS ranged from 3 to 4 in the animals. The median estimated weight was 60 (50–60) kg, and the mean actual weight was 52.2 ± 14.5 kg. All animals were weighed, except for one (D10), in which technical issues prevented completion of body weight measurement. Among the 20 deer with complete data, the estimated body weight was underestimated in 8 animals and overestimated in 12 animals. The mean difference was +5.8 kg for underestimated and −6.3 kg for overestimated animals, with an overall range from −28.8 to +15.0 kg. Based on clinical examination and hematobiochemical analyses, all animals were deemed healthy at the time of immobilization. Five females were confirmed to be pregnant.

### 3.2 Anesthesia protocol

All animals that entered the study met the inclusion criteria, completed the study without complications, and were included in the final analyses. The animals were never chased before being darted. In some cases, multiple animals were captured simultaneously. The drug combination was administered intramuscularly at different injection sites: 18 animals received the injection in the thigh, and the remaining in the shoulder. Animal D7 unintentionally received two full doses, the second of which intended for another animal, due to unexpected movement during darting. Two yearling deer (D8 and D11) were given doses intended for adults. Deer D5 and D18 were darted twice, with the first dart failing to discharge the drug after bouncing off. The mean shooting distance was 12.9 ± 2.5 meters, and the mean injection pressure was 4.9 ± 0.8 bar.

The immobilization protocol consisted of detomidine (0.2 mg/kg), ketamine (2 mg/kg), and azaperone (0.3 mg/kg), with doses based on visually estimated body weight. The drug doses were adjusted retrospectively based on actual body weight, with mean administered doses of detomidine 0.22 ± 0.07 mg/kg, ketamine 2.24 ± 0.69 mg/kg, and azaperone 0.34 ± 0.10 mg/kg.

A jugular venous catheter 14G was placed in all animals. No propofol or fluid administration was required in any case.

Not all timepoints during induction and recovery could be recorded for every animal, as some individuals were not fully visible to the observers, and in certain cases, time events overlapped. However, the time of intramuscular injection of detomidine, ketamine, and azaperone, as well as the times of sternal recumbency, approach, and atipamezole administration were consistently recorded for all subjects.

During induction, ataxia and lateral recumbency times were recorded for 13 and 10 animals, respectively. Ataxia, sternal recumbency, lateral recumbency and approach times were 4.0 ± 2.2 min, 5.2 ± 1.9 min, 10.7 ± 6.4 min, and 22.0 ± 5.1 min, respectively.

Handling time, from approach to atipamezole administration, was 49.8 ± 9.6 min.

Atipamezole was administered intramuscularly in the thigh to all animals. Times to first voluntary movement, sternal recumbency, and standing position were recorded in 2, 9, and 8 animals, respectively. Median times (interquartile ranges) were 3.5 min (2.3–4.8), 3.0 min (3.0–8.0), and 7.5 min (5.8–9.8), respectively.

At the time of antagonist administration, seven animals exhibited signs of awakening, whereas two required active physical restraint for placement into transport crates before receiving atipamezole. Consequently, the effective body weight of these two animals was not recorded.

All animals were able to stand and maintain a stable posture within 20 min from atipamezole injection, except for one animal that stood up after 30 min.

### 3.3 Clinical monitoring

Clinical monitoring began at the time of animal approach (T0), was conducted continuously, and recorded every 5 min for 38 ± 4.3 min.

Almost all parameters were consistently measured at all timepoints for all animals, except for subject D3, in which, due to instrumentation issues, it was possible to only record HR and RR every 5 min up to T40.

**Figure 1:**
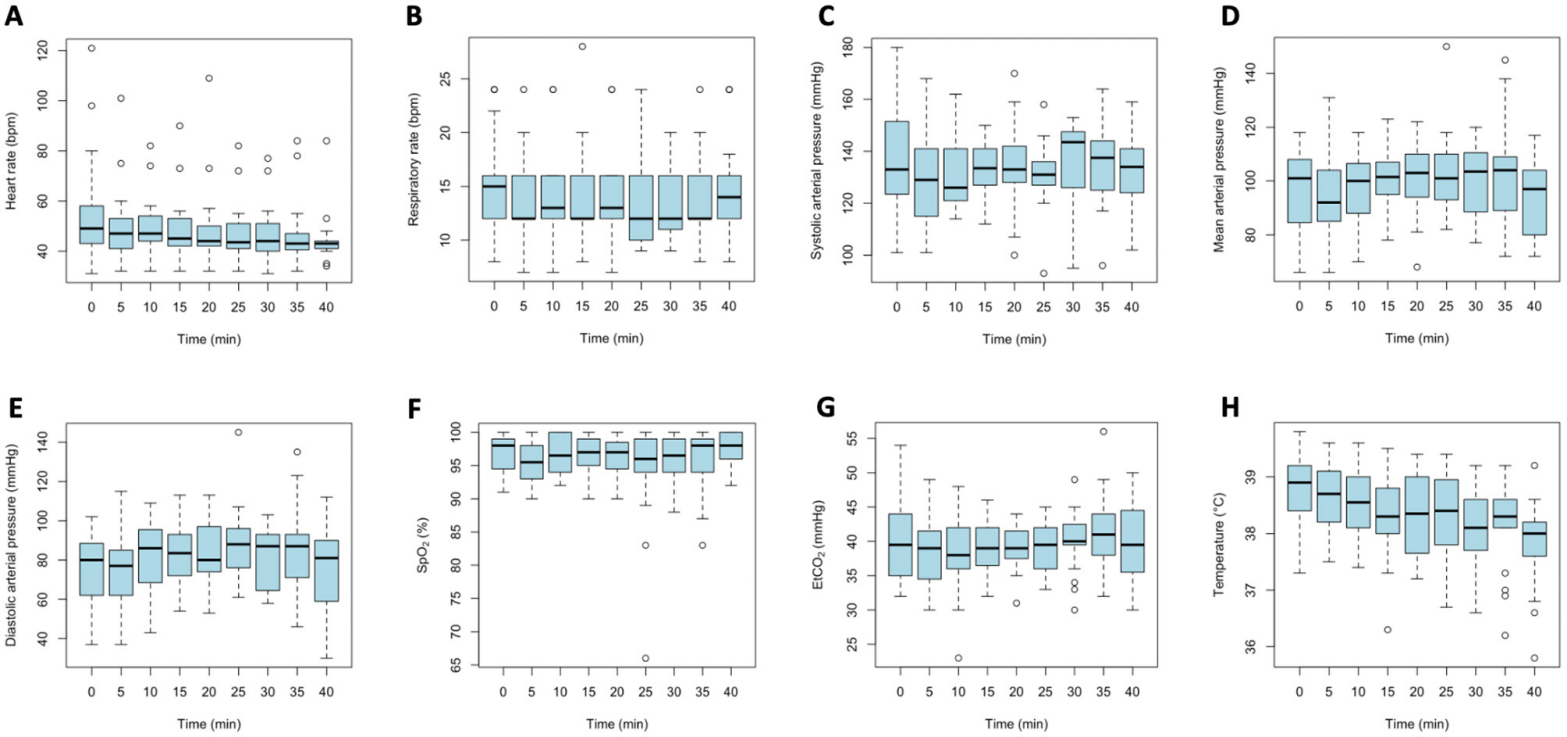
Time-course plots of physiological parameters recorded every 5 min during immobilization in fallow deer, starting from the moment of safe approach. Each panel represents a different parameter: **(A)** Heart rate; **(B)** Respiratory rate; **(C)** Systolic arterial pressure; **(D)** Mean arterial pressure; **(E)** Diastolic arterial pressure; **(F)** Peripheral oxygen saturation (SpO_2_); **(G)** End-tidal carbon dioxide (ETCO_2_); **(H)** Body temperature.

The parameters HR, RR, SpO_2_, ETCO_2_, SAP, MAP, DAP, and Temp are presented in Figure 1. Among these variables, only HR and Temp showed a significant change over time, while the others remained stable. Values at T0 were considered “baseline” values.

Specifically, for each time unit (every 5 min), there was a significant decrease in HR of approximately 0.17 bpm (*p* < 0.001). Considerable variability in HR was observed among animals' baseline values, with a standard deviation of 12.65 bpm.

Respiratory rate exhibited a slight negative trend over time (slope = −0.019), but this was not statistically significant (*t* = −1.33), indicating no significant temporal change in RR within the sample. Substantial variability was present between baseline values (standard deviation = 3.41 bpm).

Systolic arterial pressure showed a negligible and non-significant effect of time (*t* = −0.055), suggesting no systematic or relevant change during the observation period. Mild variability existed among animals' baseline values, with a baseline standard deviation of 6.65 mmHg.

Mean arterial pressure demonstrated a modest, non-significant positive trend over time (*t* = 1.359), indicating that MAP remained relatively stable throughout the observation. Mild inter-animal variability was observed (baseline standard deviation = 8.24 mmHg), with MAP consistently above 65 mmHg in all animals at all timepoints.

Diastolic arterial pressure also showed a modest and non-significant change over time (*t* = 1.733), with moderate variability between animals (baseline standard deviation = 9.73 mmHg), indicating no meaningful systematic change.

The SpO_2_ remained stable over time, with a negligible and non-significant effect of time (*t* = 0.052). Variability between animals was low (baseline standard deviation = 2.48%).

The ETCO_2_ showed a modest, non-significant temporal effect (*t* = 1.172), indicating no relevant systematic change over time. Moderate variability was seen between animals (baseline standard deviation = 2.26 units). Only two animals had single values above 50 mmHg (54 and 56 mmHg).

Body temperature demonstrated a significant and pronounced negative effect over time (*t* = −9.42), indicating a systematic decrease during the observation period. Moderate baseline variability was present among animals (standard deviation = 0.64 °C), while residual variability was lower (0.32 °C), suggesting the model effectively captured individual differences and temporal trends. Forty-seven measurements were below 38 °C, 89 ranged between 38 and 39 °C, and 43 were above 39 °C. Temperatures above 39.5 °C were recorded only at the first four time points (T0, T5, T10, or T15) in 4 animals.

Anesthesia depth was scored from 0 to 1 (Table 1) in all animals, based on palpebral and anal reflexes, muscle tone, and eye position, showing mostly absent reflexes and reduced muscle tone, indicating effective immobilization.

Lactate concentrations were measured approximately 30 min post-capture in 14 animals, with a mean value of 3.7 ± 0.7 mmol/L, while blood glucose was measured in 18 animals at about 40 min post-capture, with a mean of 159.3 ± 24.6 mg/dL.

### 3.4 Anesthesia quality assessment

Quality scores were assigned to all animals according to the criteria in Tables 2–4 and are presented in Table 5. Induction quality proved uniformly high, with 73 % of subjects rated as “excellent” and the remaining 27 % as “good”; no animal fell into the “poor” or “moderate” categories.

**Table 5:** Number of animals per score category for each scoring system: induction score (scores 0–3, see Table 2), immobilization score (scores 0–6, see Table 3), and recovery quality score (scores 0–5, see Table 4).

**Scoring system**	**Score**	**Number of animals (*n*)**
Induction score	0	0
	1	0
	2	6
	3	16
Immobilization score	0	0
	1	0
	2	0
	3	0
	4	14
	5	8
Recovery score	0	0
	1	1
	2	0
	3	2
	4	12
	5	7

Each value represents the count of animals assigned to the corresponding score category.

During immobilization, 64 % of animals exhibited a “deep” immobilization (score 4), while the other 36 % achieved “very deep” immobilization (score 5), and none showed either a more superficial or an excessively deep level.

Recovery quality was “moderate” in 54 % of cases and “excellent” in 32 %, with only two animals (D9, D14) receiving a “fair” score and a single animal (D21) experiencing a “poor” recovery.

### 3.5 Adverse effects and complications

During immobilization, rumen distention occurred in four animals (D1, D2, D5, and D13). In all cases, this was resolved through repositioning the animal in sternal recumbency and/or performing gentle ruminal massage.

Episodes of respiratory depression, defined as a RR below 10 bpm, were observed in six animals. In D6 and D20, only a single timepoint measurement fell below this threshold, with values of 9 and 8 bpm, respectively. In contrast, animals D12, D18, D19, and D22 exhibited RR below 10 at two or more timepoints, with values ranging between 7 and 9 bpm. Despite these reductions in respiratory rate, SpO_2_ remained consistently above 90% in all affected animals, and ETCO_2_ levels were below 44 mmHg.

A total of 179 SpO_2_ measurements were recorded. Seven of the measurements of SpO_2_ were below 90% (ranging from 66 to 89%) in 4 animals, however mucous membranes remained pink, and subsequent measurements in those animals were above 90%. Forty-two measurements were between 90% and 95%.

All animals recovered without observable drug-related complications or behavioral signs of distress.

No evidence of re-sedation was observed in any individual during the subsequent 6 h, and no adverse effects were noted in the following 5 days. All of the female deer successfully carried their pregnancies to term.

## 4 Discussion

The detomidine-ketamine-azaperone combination provided effective and safe immobilization in fallow deer, characterized by smooth induction, adequate anesthetic depth, and good-quality recovery in the majority of animals. This study yielded valuable clinical insights into the effects and quality of immobilization afforded by this combination, thanks to comprehensive physiological monitoring and the use of detailed scoring systems for standardized assessment. Notably, this specific drug combination has not been previously described in the scientific literature for fallow deer.

The rationale for combining an α_2_-agonist with a dissociative anesthetic such as ketamine is supported by previous studies. The use of α_2_-agonists in association with ketamine or tiletamine/zolazepam has been described in fallow deer (7, 10, 13), generally providing acceptable immobilization. Among the α_2_-agonists, detomidine offers superior receptor selectivity compared to xylazine (11), but its use in fallow deer remains poorly documented. In the present study, detomidine was administered at 0.2 mg/kg, consistent with the dose reported by Galka et al. (7) in fallow deer. The ketamine dose (2 mg/kg) was slightly higher than in the protocol described by Avni-Magen et al. (13), where the mean immobilization time was only approximately 19 ± 3 min. However, it was lower than doses reported in other studies (7, 14). Despite this relatively low ketamine dose, anesthetic depth was adequate, likely enhanced by the synergistic effect of azaperone. Unlike α_2_-agonists and dissociative anesthetics, azaperone has rarely been studied in fallow deer and has previously been reported only in combination with fentanyl or thiafentanil (9, 15). Notably, Lapid et al. (9) evaluated azaperone exclusively in Persian fallow deer (*Dama dama mesopotamica*), a subspecies distinct from the European fallow deer. Azaperone has also been infrequently reported in deer (16, 17).

This butyrophenone drug, widely used for tranquilization in swine, has also been used in wild and farmed ungulates when combined with α_2_-agonists and opioids (5). In pigs, it has a reported duration of action of approximately 6 h, though its duration in wildlife remains unclear. In this study, the azaperone dose (0.3 mg/kg) was higher than that used by Lapid et al. (9), who administered 0.190 ± 0.030 mg/kg with thiafentanil, achieving immobilization for only 21.8 ± 5.2 min. Notably, Lapid et al. (9) also reported five perianesthetic morbidity and mortality events, including four cases of respiratory arrest approximately 10 min after drug administration. These adverse effects may have been related to the use of thiafentanil, a potent opioid known for its rapid onset and potential for respiratory depression. Lapid et al. (9) also reported a more uniform immobilization score with azaperone-thiafentanil than with etorphine-acepromazine, suggesting azaperone may provide more predictable immobilization quality.

In this study, the inclusion of azaperone may have contributed to the observed smooth induction, stable anesthesia, and satisfactory recovery, supporting its potential role in cervid anesthesia protocols. Moreover, its vasodilatory properties may have helped mitigate α_2_-agonist-induced hypertension (18, 19), further improving hemodynamic stability. Importantly, five pregnant females were successfully anesthetized and carried their pregnancies to term. This suggests that, despite the theoretical risk of α_2_-agonists affecting uterine perfusion (20, 21), the protocol was safe in these cases. Notably, detomidine is known not to affect reproductive organ perfusion while exerting typical cardiovascular effects (22). This supported its use in this study.

The results of this study also highlight the robustness and wide safety margin of the detomidine-ketamine-azaperone protocol. No complications were observed in this study, although some animals received drug doses that exceeded the calculated target amount, with a few receiving up to twice the intended dose. In chemical immobilization of wildlife, several factors may commonly affect dosing accuracy, including estimated body weight, dart volume limitations, and challenges associated with drug delivery under field conditions. For this reason, it is essential that pharmacological protocols offer a wide safety margin.

Another positive aspect of the protocol used in this study is the excellent quality of induction observed in the vast majority of animals (73%), and good in the remainder. Induction time is a critical factor in wildlife immobilization, as it helps to minimize stress and reduce the risk of trauma (23). In this study, mean times to ataxia (4.0 ± 2.2 min), sternal recumbency (5.2 ± 1.9 min), and lateral recumbency (10.7 ± 6.4 min) were recorded, with an average approach time of 22.0 ± 5.1 min.

Comparing the approach time used in this study to the induction times reported in other studies, these values are comparable to, or slightly longer than, those described in protocols for other cervids (7, 10, 13).

However, it is important to note that the approach time in this study reflects the actual physical approach of the operators, which was intentionally delayed to reduce stress in confined group settings. This differs from induction time reported in the literature, which usually refers to the interval from drug administration to loss of posture or recumbency. Previous research recommends waiting at least 15 min before approaching sedated wildlife, to allow full drug effect (24), as drug absorption after intramuscular injection is influenced by muscle perfusion and cardiac output. The onset of drug action varies with the agent used: approximately 15 min for α_2_-agonists, 1–2 min for ketamine, and around 10 min for azaperone (11). Loss of standing posture is considered a reliable indicator of deepening sedation, especially when associated with signs like head lowering, decreased respiratory rate, and reduced responsiveness. However, even when recumbent, animals may not yet be fully sedated, and early stimulation may trigger arousal or escape attempts. This reaction is commonly associated with α_2_-agonists and may be due to sudden stress-induced catecholamine release that counteracts sedation (24). Therefore, delaying approach after recumbency is generally advised. Despite these variations, no adverse events related to induction occurred in this study. All animals lost standing posture rapidly after darting, which effectively minimized the risk of injury or escape, and highlights the reliability and safety of the detomidine-ketamine-azaperone protocol under field conditions.

Immobilization quality was also consistently high, ranging between deep and very deep. Adequate anesthetic depth was confirmed by the absence of muscle tone, preserved relaxation, and a lack of nociceptive responses during painful procedures. The occasional presence of weak palpebral reflexes did not compromise the safety or adequacy of immobilization. Notably, no reactions were observed during venipuncture, microchip implantation, or ear tagging, indicating that the drug combination provided effective analgesia for minor procedures. This is consistent with the known, albeit short-lasting, analgesic effects of α_2_-agonists (11).

Continuous monitoring of vital signs during chemical immobilization revealed good cardiopulmonary stability throughout. Heart rates remained within expected ranges for α_2_-agonist/dissociative protocols (7, 10, 13) and were generally lower than those reported with opioid-based combinations (9, 25). A statistically significant decrease in heart rate was observed during anesthesia, consistent with other studies involving α_2_-agonists in other animal species (26, 27). However, the reduction (approximately 0.17 bpm every 5 min) was very minimal and not clinically relevant. Importantly, this decrease in heart rate was not associated with changes in blood pressure, which remained stable and within physiological limits for cervids sedated with similar protocols (9, 13). The inclusion of azaperone likely contributed to cardiovascular stability by attenuating detomidine-induced hypertension through peripheral vasodilation mediated by dopaminergic and α1-adrenergic antagonism (18). This balancing effect has also been reported in other cervid species when α_2_-agonists are combined with azaperone (19, 28), supporting its use in improving cardiovascular safety during immobilization. Mild variability in baseline heart rate and blood pressure values was noted among individual animals at the start of monitoring, likely reflecting differences in individual responses, stress levels, or temperament.

Throughout the immobilization, respiratory parameters also remained within acceptable clinical limits, indicating good physiological stability under the detomidine-ketamine-azaperone protocol. The SpO_2_ values showed a stable and non-significant trend over time. Similarly, ETCO_2_ exhibited a modest, non-significant upward trend, suggesting an absence of progressive hypoventilation or accumulation of CO_2_. Although two isolated values exceeded 50 mmHg (54 and 56 mmHg), these variations were not associated with any clinical signs of concern. Respiratory depression, defined as a respiratory rate below 10 breaths per minute, was observed in six animals. In two individuals, this occurred only at a single time point, while four animals displayed RR values between 7 and 9 bpm at multiple time points. Despite these reductions, SpO_2_ remained above 90% in all affected animals, and ETCO_2_ did not exceed 44 mmHg, suggesting that ventilation was still adequate and that hypoventilation did not progress to clinically significant hypoxia or hypercapnia. These findings are encouraging, particularly given that α_2_-agonists are known to cause reductions in respiratory rate in ruminants (29, 30). A total of seven SpO_2_ measurements fell below 90%, ranging from 66% to 89%, in four individuals. However, mucous membranes remained pink, and SpO_2_ values normalized in subsequent readings. Moreover, 42 measurements (across different animals and time points) were within the 90–95% range. Supplemental oxygen was intended to be administered via flow-by in cases where SpO_2_ dropped below 90% and RR was < 10 bpm. As no species-specific thresholds are available for fallow deer and the animals were breathing spontaneously, with no possibility to measure tidal volume or arterial blood gases, conservative criteria were applied to define respiratory depression in order to ensure animal safety.

Much lower SpO_2_ and RR values were frequently reported in similar species under different immobilization protocols (9, 13). Notably, the accuracy of pulse oximetry in fallow deer remains uncertain. In addition, it has been shown that SpO_2_ may underestimate arterial oxygen saturation (SaO_2_), due to poor agreement between these parameters in various wild species (31). This limitation may be further exacerbated by the use of α_2_-agonists, which can impair peripheral perfusion and pulse oximeter signal quality in animals (31, 32). Moreover, it is well known that the ability of pulse oximeters to accurately read SpO_2_ is influenced by the site of probe placement (32, 33). However, in the absence of direct blood gas analysis, which was not feasible in this study due to logistical and economic constraints, pulse oximetry provided a useful, albeit indirect, tool for monitoring respiratory adequacy. Regardless of the results of specific monitoring, supplemental oxygen is strongly recommended during field immobilization of cervids (34). Indeed, α_2_-agonist-induced reductions in arterial oxygen tension have been documented in sheep, highlighting the importance of oxygen support during sedation in ruminants (35). In this study, however, supplemental oxygen was not routinely administered, but rather, it was available if clinically necessary. This approach was adopted to better evaluate the actual impact of the anesthetic protocol on respiratory function in the field, where access to oxygen sources is not always guaranteed.

The protocol did not cause hyperthermia in any animal. In the study by Avni-Magen et al. (13) involving captive Persian fallow deer immobilized with medetomidine-ketamine or medetomidine-midazolam, body temperatures remained below 40.5 °C. In contrast, Lapid et al. (9) reported severe hyperthermia in 3 out of 20 Persian fallow deer immobilized with thiafentanil-azaperone, possibly due to muscle rigidity, a condition not observed in our study. On the contrary, we recorded a decrease in temperature over time, which is a common physiological response during anesthesia. Anesthetic agents, including sedatives, and α_2_-agonists, are known to widen the interthreshold range for thermoregulation, reducing the animal's ability to mount compensatory responses such as shivering (36).

Metabolic evaluation was limited but informative as only single-point lactate and glucose measurements could be collected due to the nature of the procedures. No cases of hypoglycemia or significant hyperglycemia were observed, despite the known hyperglycemic effects of α_2_-agonists (37), suggesting that the animals were not experiencing severe stress or excessive sympathetic stimulation. Lactate levels exceeded 2.5 mmol/L in several animals but remained below values reported in previous studies involving fallow deer immobilization (9), further supporting the notion of stable cardiovascular and metabolic status under the protocol used. Elevated lactate is commonly associated with increased muscular activity during capture and handling, which can also lead to hyperthermia, muscle enzyme leakage, and lactic acidosis. If uncompensated, these changes may progress to a clinical syndrome characterized by shock, myopathy, renal failure, muscle rupture, or even sudden death (24). The lactate values observed, together with the absence of significant increases in body temperature, suggest that the capture method and anesthetic protocol employed did not promote the pathophysiological cascade typically associated with capture myopathy. Future studies should evaluate these values at a minimum of two time points, immediately after the initial approach and prior to release, to provide a more accurate understanding of the effects of the protocol and handling on these parameters.

Overall, the cardiovascular, respiratory, and metabolic findings in this study support the conclusion that the detomidine-ketamine-azaperone combination provides a high degree of cardiopulmonary safety, with only minimal and clinically irrelevant respiratory depression and no evidence of significant metabolic derangement. Additionally, careful observation and timely interventions, such as repositioning animals and performing gentle ruminal massage, proved effective in managing ruminal distention when it occurred. This condition was observed in only four animals, despite being a well-documented side effect of chemical immobilization in polygastric herbivores with α_2_-agonists (30, 38, 39). The reduction in gastro-intestinal motility associated with α_2_-agonists is believed to result from the suppression of gastrointestinal regulatory centers within the medulla oblongata (40, 41). The low incidence and successful management of this complication further underscore the practical safety and field applicability of the protocol.

In this study, atipamezole was administered at a 1:1 ratio with detomidine. Recommended atipamezole doses vary by species and α_2_-agonists. Furthermore, the efficacy of antagonism depends on the sedative dose, time, and route of administration (11). Moreover, there are no specific studies evaluating the effective dose of atipamezole required to antagonize detomidine in cervids based on the time elapsed since drug administration. Notably, previous studies have successfully used a 1:1 ratio with antelopes and sheep (42, 43).

In the present study, no fallow deer exhibited clinical signs of re-sedation during the 6-h post-atipamezole monitoring period. This observation period was selected as a precautionary measure, since the pharmacokinetics of α_2_-agonists and their antagonism by atipamezole are not well established in fallow deer. Considering that atipamezole has a shorter half-life (approximately 1.5–2 h) than most α_2_-agonists (11), extended monitoring was deemed necessary to detect potential re-sedation or delayed adverse effects and to ensure animal welfare. Moreover, as field conditions allowed observation only under natural daylight, a 6-h period was deemed appropriate to ensure reliable monitoring and safeguard animal welfare. Based on our findings, it is reasonable to assume that no re-sedation occurred beyond this timeframe.

The safety of the protocol was further supported by the quality and rapidity of recovery observed in this study. Most animals exhibited moderate to excellent recovery quality, with only one individual classified as poor. Nonetheless, all animals were able to regain a standing position within 20 min post-procedure. These findings indicate that the detomidine-ketamine-azaperone combination allows for smooth and predictable recoveries, minimizing the risk of post-anesthetic complications and facilitating handling and safe release in field conditions. Similar recovery times were also reported by Avni-Magen et al. (13). In contrast, Lapid et al. (9) observed shorter recovery times; however, the quality of immobilization was inferior and several complications were noted.

However, certain limitations of this study must be acknowledged. The lack of a control or comparison group prevents direct evaluation against other commonly used protocols. The limited sample size, particularly across age classes, prevented subgroup analyses regarding dose effects or age-related sensitivity. Since the study was conducted on animals accustomed to human presence, the results may not be generalizable to free-ranging populations which could exhibit heightened stress responses. Additionally, all captures occurred under temperate conditions. Further studies are needed to evaluate the influence of environmental variables such as ambient temperature or seasonal changes on sedation and recovery quality. Testing the protocol under more challenging conditions, including in wild or free-ranging deer, would further assess its robustness.

## 5 Conclusions

The detomidine-ketamine-azaperone combination demonstrated excellent performance for the chemical immobilization of fallow deer, providing smooth induction, stable anesthesia, and good to excellent recovery quality in most animals. The protocol showed a favorable safety profile and represents a reliable, non-opioid option for routine clinical and management procedures in this species. While azaperone has previously been described in fallow deer in combination with thiafentanil or fentanyl, this is the first study to evaluate its use alongside detomidine and ketamine, highlighting its potential as a valuable adjunct in cervid anesthesia protocols. Further research is warranted to confirm these findings and optimize dosing strategies.

## Data Availability

The raw data supporting the conclusions of this article will be made available by the authors, without undue reservation.
